# The Alzheimer's-related amyloid beta peptide is internalised by R28 neuroretinal cells and disrupts the microtubule associated protein 2 (MAP-2)

**DOI:** 10.1016/j.exer.2016.10.013

**Published:** 2016-12

**Authors:** George Taylor-Walker, Savannah A. Lynn, Eloise Keeling, Rosie Munday, David A. Johnston, Anton Page, Jennifer A. Scott, Srini Goverdhan, Andrew J. Lotery, J. Arjuna Ratnayaka

**Affiliations:** a Clinical and Experimental Sciences, Faculty of Medicine, University of Southampton, SGH, MP806, Tremona Road, Southampton, SO16 6YD, United Kingdom; b Biomedical Imaging Unit, University of Southampton, SGH, MP12, Tremona Road, Southampton, SO16 6YD, United Kingdom; c Eye Unit, University Southampton NHS Trust, Southampton, SO16 6YD, United Kingdom

**Keywords:** Amyloid beta (Aβ), Neuroretina, R28 cells, Retinal degeneration, MAP-2, AD, Alzheimer's disease, BrM, Bruch's membrane, GA, Geographic atrophy, MAP-2, Microtubule associated protein-2, nM, Nanomolar, Nv, Neovascular, PBS, Phosphate buffered saline, PFA, Paraformaldehyde, PKC, Protein kinase C, RGC, Retinal ganglion cells, RPE, Retinal pigment epithelium, TEM, Transmission electron microscopy, VEGF, Vascular endothelial growth factor

## Abstract

Age-related Macular Degeneration (AMD) is a common, irreversible blinding condition that leads to the loss of central vision. AMD has a complex aetiology with both genetic as well as environmental risks factors, and share many similarities with Alzheimer's disease. Recent findings have contributed significantly to unravelling its genetic architecture that is yet to be matched by molecular insights. Studies are made more challenging by observations that aged and AMD retinas accumulate the highly pathogenic Alzheimer's-related Amyloid beta (Aβ) group of peptides, for which there appears to be no clear genetic basis. Analyses of human donor and animal eyes have identified retinal Aβ aggregates in retinal ganglion cells (RGC), the inner nuclear layer, photoreceptors as well as the retinal pigment epithelium. Aβ is also a major drusen constituent; found correlated with elevated drusen-load and age, with a propensity to aggregate in retinas of advanced AMD. Despite this evidence, how such a potent driver of neurodegeneration might impair the neuroretina remains incompletely understood, and studies into this important aspect of retinopathy remains limited. In order to address this we exploited R28 rat retinal cells which due to its heterogeneous nature, offers diverse neuroretinal cell-types in which to study the molecular pathology of Aβ. R28 cells are also unaffected by problems associated with the commonly used RGC-5 immortalised cell-line, thus providing a well-established model in which to study dynamic Aβ effects at single-cell resolution. Our findings show that R28 cells express key neuronal markers calbindin, protein kinase C and the microtubule associated protein-2 (MAP-2) by confocal immunofluorescence which has not been shown before, but also calretinin which has not been reported previously. For the first time, we reveal that retinal neurons rapidly internalised Aβ_1-42_, the most cytotoxic and aggregate-prone amongst the Aβ family. Furthermore, exposure to physiological amounts of Aβ_1-42_ for 24 h correlated with impairment to neuronal MAP-2, a cytoskeletal protein which regulates microtubule dynamics in axons and dendrites. Disruption to MAP-2 was transient, and had recovered by 48 h, although internalised Aβ persisted as discrete puncta for as long as 72 h. To assess whether Aβ could realistically localise to living retinas to mediate such effects, we subretinally injected nanomolar levels of oligomeric Aβ_1-42_ into wildtype mice. Confocal microscopy revealed the presence of focal Aβ deposits in RGC, the inner nuclear and the outer plexiform layers 8 days later, recapitulating naturally-occurring patterns of Aβ aggregation in aged retinas. Our novel findings describe how retinal neurons internalise Aβ to transiently impair MAP-2 in a hitherto unreported manner. MAP-2 dysfunction is reported in AMD retinas, and is thought to be involved in remodelling and plasticity of post-mitotic neurons. Our insights suggest a molecular pathway by which this could occur in the senescent eye leading to complex diseases such as AMD.

## Highlights

• Molecular basis of complex retinopathies such as AMD is incompletely understood.
• The Alzheimer's-related Aβ peptides are rapidly internalised by retinal neurons.
• Internalised Aβ is retained within neurons and transiently impairs MAP-2.
• Subretinally injected Aβ mimics its naturally-occurring distribution in aged retinas.

## Graphical abstract

**Figure:**
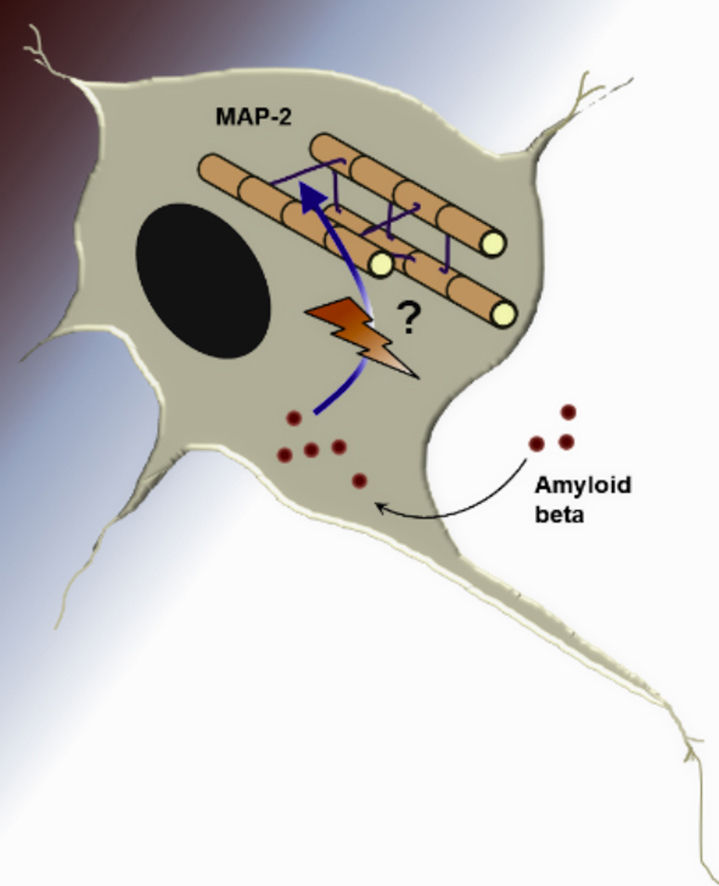

## 1 Introduction

Age-related Macular Degeneration (AMD) is a common blinding condition that leads to the irreversible loss of central vision amongst the elderly (Khandhadia et al., 2012, Lotery, 2008). The disease manifests from midlife onwards to affect over ½ million individuals in the UK (source: Macular Society, UK), or approximately 50 million individuals globally (Gordois et al., 2012). The current strategy of using anti-vascular growth factor (VEGF) inhibitors to treat the less common neovascular (nv) form is inadequate, as prolonged treatment appears to damage the remaining retinal pigment epithelium (RPE) leading to the geographic atrophy (GA) form of AMD (Grunwald et al., 2015, Lois et al., 2013). Whilst this treatment has benefited many nvAMD patients by restoring partial sight, its limited effect reveals the restrictions of a therapy based on an incomplete understanding of this complex disease. Moreover, it is almost impossible to maintain initial visual gains seen with anti-VEGF therapy due to the need for indefinite treatment in some patients (Hykin et al., 2016). The prognosis for GA patients is considerably worse, as there is currently no treatment (Khandhadia et al., 2012). Recent advances have contributed substantially to revealing the genetic basis of AMD (Fritsche et al., 2013, Fritsche et al., 2016). However, AMD, like other complex chronic degenerative conditions such as Alzheimer's disease (AD), cannot solely be defined by genetics (Sanchez-Mut and Graff, 2015, Sato and Morishita, 2013). For instance, factors such as an unhealthy life-style, poor diet and obesity are all known to play considerable roles in increasing the risks of sight loss (Chiu et al., 2014, Khandhadia et al., 2012, Pikuleva and Curcio, 2014). To add to this complexity, the Alzheimer's-associated Amyloid beta (Aβ) group of misfolding proteins were reported to accumulate in ageing retinas (Ratnayaka et al., 2015). Such Aβ deposits were found in retinal whole-mounts associated with photoreceptor outer segments, the RPE and Bruch's membrane (BrM), as well as in choroidal vessels (Hoh et al., 2010, Koronyo-Hamaoui et al., 2011). Increased retinal Aβ loads were also correlated with advancing age and with high levels of drusen (Anderson et al., 2004). Of note, Aβ was reported to be within drusen, organised into spheres referred to as amyloid vesicles (Anderson et al., 2004, Johnson et al., 2002). Ultrastructural and confocal immunofluorescence analysis revealed these vesicles to have a concentric ring-like interior permeated with Aβ immunoreactivity (Anderson et al., 2004). Such vesicles also formed a substantial proportion of drusen volume (Anderson et al., 2004, Isas et al., 2010, Luibl et al., 2006). It appears that retinal Aβ is correlated with advanced forms of AMD (Anderson et al., 2004), with one study reporting Aβ-positive drusen only in patients with AMD (Dutescu et al., 2009). Furthermore, well-established AMD risk factors such as cholesterol and high-fat diets are also associated with increased retinal Aβ loads (Kauppinen et al., 2016, Khandhadia et al., 2012, Ratnayaka et al., 2015). This pattern of Aβ aggregation suggests that the RPE, the focus of most AMD pathology, may be particularly at risk from Aβ. However, Aβ also appears to accumulate in specific regions of the neuroretina. For example, Aβ immunoreactivity was reported in retinal ganglion cells (RGC) of mice (Guo et al., 2007) and in the inner nuclear layer and RGC of rabbits (Dasari et al., 2011), whilst curcumin positive Aβ plaques were observed in retinal whole-mounts of AD patients (Koronyo-Hamaoui et al., 2011). This was not surprising as some retinal neurons express the amyloid precursor protein (APP) (Dutescu et al., 2009, Wang et al., 2011); the precursor form from which Aβ is cleaved (Benilova et al., 2012). Aβ_1-42_, which is considered to be the most pathogenic and aggregate-prone of the Aβ species (Citron et al., 1997), was shown to increase in RGC with increasing age (Wang et al., 2011). This observation is consistent with evidence that senescent RPE also produce increasing levels of Aβ (Yoshida et al., 2005). Despite such indications there is very little understanding of how Aβ can actually affect retinal neurons. Key questions such as how Aβ behaves in the retina and what its long-term effect may be remains unanswered. Here, by utilizing the rat neuroretinal R28 cells (Seigel, 1996), we assessed how Aβ might affect the neuroretina at single-cell resolution. Our findings reveal for the first time that oligomeric Aβ_1-42_ is rapidly internalised by retinal neurons. Furthermore, exposure to nanomolar (nM) quantities of Aβ resulted in the transient downregulation of microtubule associated protein-2 (MAP-2). Our findings also show that retinal neurons retained internalised Aβ long after initial exposure. We also show for the first time that subretinally injected Aβ_1-42_ accumulates in the neuroretina mimicking naturally-occurring patterns of Aβ deposition, and indicating that these have the potential to realistically impair retinal MAP-2. Our findings provide new insights into how Aβ can target the neuroretina, contributing to chronic degenerative conditions such as AMD.

## 2 Materials and methods

### 2.1 Preparation and characterisation of Aβ

Human recombinant lyophilized Aβ_1-42_ 1,1,1,3,3,3-hexafluoro-2-propanol (HFIP) was purchased from rPeptide (Bogart, GA, USA) for use in experiments. Lyophilized product was solubilized to 1 mg/mL in HFIP (Sigma, UK), vortexed for 1 min and sonicated for 5 min in a 50 Hz FS100 Frequency Sweep (Decon, UK) bath sonicator to ensure proper reconstitution of samples. Evaporation of HFIP was performed using dry nitrogen and subsequent vacuum desiccation of samples for 30 min to remove residual resuspension solvent. The resultant Aβ_1-42_ peptidic film was resuspended in DMSO (ACROS Organics, US) to 1 mg/mL, vortexed and allowed to stand for 1 min before being added to a 2 mL Zeba desalting column, equilibrated with Aβ buffer (10 mM HEPES, 50 mM NaCl, 1.6 mM KCl, 2 mM MgCl_2_·6H_2_O and 3.5 mM CaCl_2_·2H_2_O pH 7.4) to facilitate buffer exchange. 40 μL of buffer was applied as a stack and the columns spun at 1000 g for 2 min at 4 °C to collect eluates containing Aβ_1-42_ peptide. Samples were subsequently spun at 16,000 g for 30 min in a 4 °C-controlled centrifuge to pellet small amounts of unwanted higher molecular weight aggregates, whilst the supernatant containing monomeric Aβ was harvested and incubated on ice for 1½ h prior to use in experiments. Peptide concentrations were calculated from the absorbance measured with a Nano drop ND-100 at 280 nm as well as the Aβ molar co-extinction efficient of 1490 M^−1^ cm^−1^ using Beer Lamberts Law. Typical Aβ yields were between 70 and 120 μM, which correspond to Aβ concentrations reported by others (Broersen et al., 2011). A similar technique was used to prepare Alexa Fluor^®^ 647-tagged Aβ_1-42_. In this case, Aβ_1-42_ peptides in DMSO were pre-incubated with 20 μl of 1 M sodium bicarbonate and 10 μl of 11.3 nm/μl dye in ddH_2_O for 15 min at room temperature prior to loading into buffer exchange columns. Concentrations of Alexa Fluor^®^ 647-tagged Aβ_1-42_ preparations were determined as before; accounting for the contribution of the dye to absorbance readings at 280 nm, as per manufacturer's instructions (Invitrogen, UK). Lo-bind Eppendorf tubes and tips were used during experiments to achieve maximal harvest (Soura et al., 2012).

### 2.2 Cell culture

R28 cells (Seigel, 1996) (Kerafast Inc., MA, USA) were cultured as described (Seigel, 2014) in a medium consisting of Dulbecco's modified eagle's medium (ThermoFisher, UK), 10% foetal calf serum (FCS) (Sigma, UK), 0.37% NaCO_3_, 0.058% l-glutamine and 100 μg/ml gentamycin (Sigma-Aldrich, UK). Cells were maintained in a humidified incubator at 37 °C and 5% CO_2_. Cultures were monitored daily by phase-contrast light microscopy to ensure that cells were maintained at sub-confluent levels. All experiments were carried out between passages 6–22. For confocal immunofluorescence experiments, cells were seeded on 100 μg/ml laminin (Sigma, UK) coated coverslips at sub-confluent levels and experiments performed within 2–5 days. Cultures were incubated with equal volumes of either human oligomeric Aβ_1-42_ (final concentration of Aβ in each well equated to 1000 nM) or vehicle. Coverslips were fixed at 24, 48 and 72 h with 4% paraformaldehyde (PFA) (Sigma, UK) for 15 min at 4 °C.

### 2.3 Animals

All aspects of animal studies were carried out in accordance with the U.K Animals (Scientific Procedures) Act, 1986. Furthermore, animal studies complied with ARRIVE guidelines, ethical oversight of the host institution's Local Research Committee and adhered to the statement for use of animals in ophthalmic and vision research by the Association for Research in Vision and Ophthalmology (ARVO). Male and female C57BL/6 mice were sourced from the Biomedical Research Facility (University of Southampton, UK), maintained on a standard 12 h light/dark cycle and allowed access to water and food *ad libitum*. Animals aged between 4 and 5 months were anaesthetised intraperitoneally with ketamine (6 mg/ml) and dexmedetomidine (0.5 mg/ml). Pupils were dilated with 1% tropicamide and 2.5% phenylephrine drops. Mice were placed under a surgical microscope and subretinally injected using a sharp 34 gauge needle with either 625 nM human oligomeric Aβ_1-42_ (n = 10 eyes from 10 separate animals) or vehicle (n = 3 eyes from 3 separate animals) in a 2 μl volume. These numbers exclude mice that developed occasional retinal bleeds following subretinal injection. Animals were revived using 0.5 mg/ml intraperitoneal anti-sedan. Soon after injections, funduscopic images confirmed the presence of subretinal blebs. At 8 days post injection, mice were anesthetised as before and the fundus imaged prior to animals being killed.

### 2.4 Histological analysis

Mice were euthanized on day 8 following exposure to Aβ_1-42_ or vehicle. Eyes were enucleated within 5 min of euthanasia and were immediately fixed in 4% PFA for 30 min at 4 °C, followed by washing in 1× phosphate buffered saline (PBS) and dissection to remove the anterior portion and lens. Samples were then processed through a series of sucrose gradients to reduce water content via osmotic potential before being embedded in optimal cutting temperature formulation. Serial cryosections of samples at 16 μM intervals were obtained using a Leica CM1850 UV microtome (Leica Microsystems, UK) which were collected on superfrost^®^ plus glass microscope slides (Thermo Scientific, UK). Sections were then probed with the appropriate set of primary and secondary antibodies.

### 2.5 Immunofluorescence and confocal microscopy

Sub-confluent and confluent R28 cells fixed with 4% PFA on coverslips were permeabilised and blocked with 1% bovine serum albumin (BSA) supplemented with 0.1% Triton X-100 (Sigma-Aldrich, UK) for 45 min at room temperature. Cultures were probed with the following primary antibodies. Mouse anti-calretinin 6B8.2 (1:200, Millipore, UK), rabbit anti-PKC-α (1:500, Abcam, UK), rabbit anti-β3-tubulin (1:200, Abcam, UK), rabbit anti-calbindin D-28K (1:500, Millipore, UK), rabbit anti-MAP-2 (1:200, Abcam, UK) and mouse monoclonal anti-human Aβ 82E1 antibody (1:100; IBL, Japan). Secondary antibodies were goat anti-mouse 488 or 594 (1:200, Invitrogen, UK), goat anti-rabbit 488 or 594 (1:200, Invitrogen, UK) and cytopainter phalloidin iFluor 647 (1:1000, Abcam, UK). Direct visualisation of Aβ was achieved via an Alexa Fluor^®^ 647-tagged motif. Cryosectioned mouse retinal tissues on glass coverslips were washed and coated in blocking serum (10% normal goat serum, 0.3% Triton X-100 in PBS) for 1 h at room temperature. Tissues were probed overnight with rabbit anti-β3-tubulin (1:200, Abcam, UK) and mouse monoclonal anti-human Aβ 82E1 antibody (1:100; IBL, Japan) followed by secondary antibodies goat anti-rabbit 594 (1:200, Invitrogen, UK) and goat anti-mouse 488 (1:200, Invitrogen, UK) for 1 h at room temperature. Cell nuclei were counterstained with 1 μg/ml of 4′,6′-diamino-2-phenylindole (DAPI) in all cases. R28 cells on coverslips as well as mouse retinal tissues were embedded in Moweol mounting medium with Citifluor anti-fade between two glass coverslips. Images were acquired using a Leica SP8 laser-scanning confocal microscope (Leica Microsystems, UK). Dynamic information within confocal images remained unaltered in subsequent image manipulations in Leica or Photoshop software.

### 2.6 Transmission electron microscopy

Negative stain transmission electron microscopy (TEM) was performed on Aβ_1-42_ preparations to visualise their assembly. 5 μl samples were applied to formvar/carbon coated 200 mesh copper grids (Agar Scientific, UK) at 0, 1, 1½, 3, 24 and 48 h post preparation for 5 s and blotted dry. 5 μl of negative stain comprising 3% ammonium molybdate in 0.1 M ammonium acetate buffer (pH 7.0) with 1 grain of sucrose/mL was subsequently applied to grids for 10 s and immediately blotted dry. Grids were allowed to air dry prior to visualising samples on a Hitachi H7000 microscope (Hitachi High Technology, Japan) fitted with a SIS Megaview III plate camera (EMSIS, Germany). We also visualised Aβ assembly using immunogold labelling experiments. Here, 5 μl samples were applied to grids for 5 min, rinsed in wash buffer (0.1 M phosphate buffer with 1% BSA) and labelled with a mouse monoclonal 82E1 antibody (1:100; IBL, Japan) for 1 h at room temperature. Unbound antibodies were removed by 3× rinses in wash buffer prior to incubation with a goat anti-mouse immunogold-conjugated secondary antibody (1:50; Elektron Technology, UK). Following incubation, samples were rinsed an additional 3× in wash buffer and once in distilled water before being blotted dry. All antibody incubations were performed in 0.1 M PBS with 1% BSA at room temperature. Negative stains of immunogold labelled grids were performed as described earlier.

### 2.7 Statistical analysis

Statistical analysis was performed using GraphPad Prism (GraphPad, USA). Data were subject to the unpaired student's *t*-test to compare total fluorescence readouts from R28 cells treated with 1000 nM Aβ_1-42_ (n = 12) vs. cultures treated with vehicle only (n = 10) to assess whether Aβ is capable of inducing alterations to MAP-2. Data is shown as mean values ± standard error of the mean (SEM) where a statistical significance of p ≤ 0.05 is denoted by a single asterisk.

## 3 Results

### 3.1 Establishing timelines for in-vitro extraction of oligomeric Aβ

**Fig. 1:**
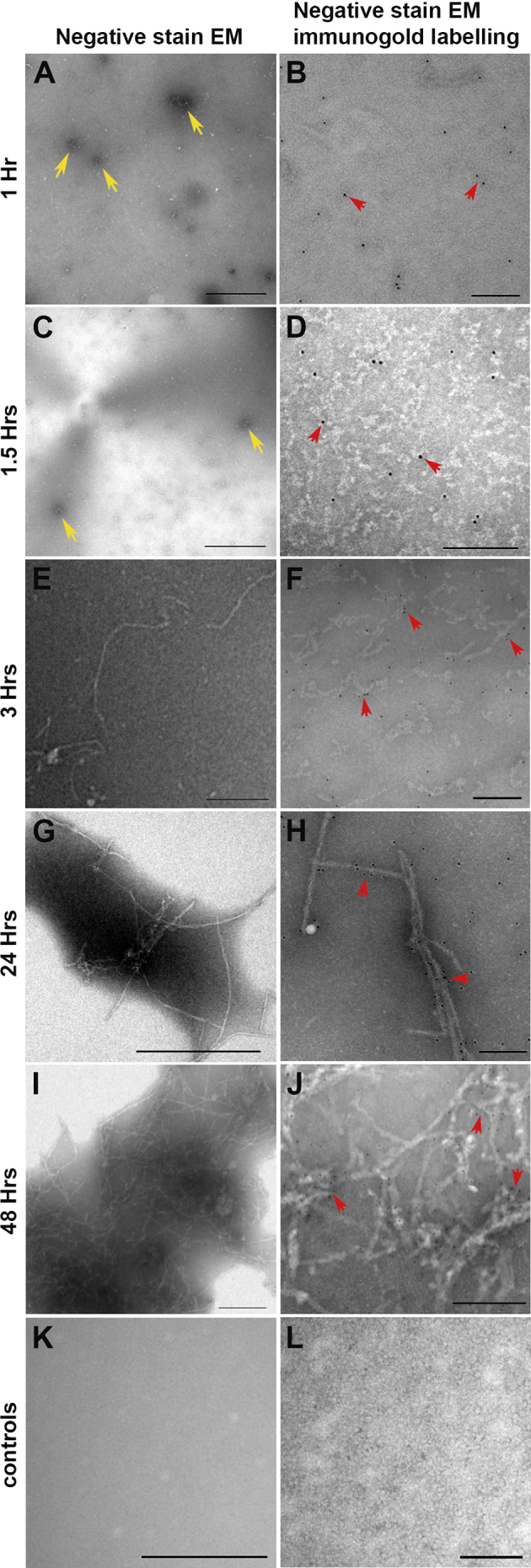
The in-vitro aggregation dynamics of human Aβ_1-42_. Transmission electron micrographs showing aggregation of negatively stained and immunogold labelled human Aβ_1-42_ at 1, 1½, 3, 24 and 48 h. [A, C] Small amorphous aggregates including oligomers are visible 1-1½ h after preparation, showing heavy negative staining (yellow arrows) which were [B, D] confirmed to be Aβ by the anti-Aβ specific 82E1 and immunogold labelling (red arrows). Aβ continued to aggregate to form larger structures over time including protofibrils and mature fibrils that can be observed in [E, G and I] negative stained sections (lighter negative stain surrounding larger structures) and in corresponding [F, H and J] immunogold labelled images (patterns of electron-dense gold particles surrounding fibrillar assemblies). Not all Aβ aggregated, as some remained as discrete structures or isolated immunogold-labelled puncta [E, F], indicating a degree of heterogeneity at later time points. [K] Vehicle controls showed no negative staining or [L] electron-dense particles after incubation with immunogold secondary antibody only. Scale bars correspond to 200 nm. (For interpretation of the references to colour in this figure legend, the reader is referred to the web version of this article.)

The dynamics of Aβ aggregation follows a well-established pattern of self-assembly, where monomers initially associate to form dimer, trimers and oligomers before aggregating into protofibrils and mature fibrils. Soluble oligomeric Aβ is known to be the most pathogenic; able to penetrate biomimetic membranes compared to more complex Aβ forms (Williams et al., 2010, Williams et al., 2011). Using a well-established protocol of in-vitro Aβ preparation (Broersen et al., 2011, Soura et al., 2012), we identified a suitable time point at which abundant Aβ_1-42_ oligomers could be readily isolated for our in-vitro and in-vivo experiments. Visualisation of Aβ preparations (80–110 μM) at different time points by negative stain TEM revealed discrete structures corresponding to 15–65 nm in diameter at 1 and 1½ h after preparation (Fig. 1A, C). At 3 h we observed the emergence of protofibrils (Fig. 1E), whilst fibrils of growing complexity with progressively heavier negative staining appeared at 24 and 48 h (Fig. 1G, I). No structures were observed on grids coated with vehicle (Fig. 1K). In order to confirm that these structures were Aβ, we used the 82E1 antibody that only recognises the Aβ N-terminus residues 1–16. Immunogold labelling coupled to this antibody showed electron-dense gold particles on structures corresponding to the size of oligomers between 1 and 1½ h (Fig. 1B, D), as well as regular patterns of staining along more elaborate protofibrils and mature fibrils at later time points (Fig. 1F, H, J). However, even by 24 h, not all Aβ had assembled into fibrils, indicating a certain degree of heterogeneity amongst immunogold-positive structures (Fig. 1H). No immunogold labelling was observed on grids probed with the secondary antibody only (Fig. 1L). Based on this pattern and rate of Aβ aggregation, oligomeric forms were observed to be most prevalent approximately 1½ h after preparation. We therefore harvested oligomeric Aβ_1-42_ at this critical time point for subsequent in-vitro and in-vivo studies.

### 3.2 R28 cells express important neuronal markers

**Fig. 2:**
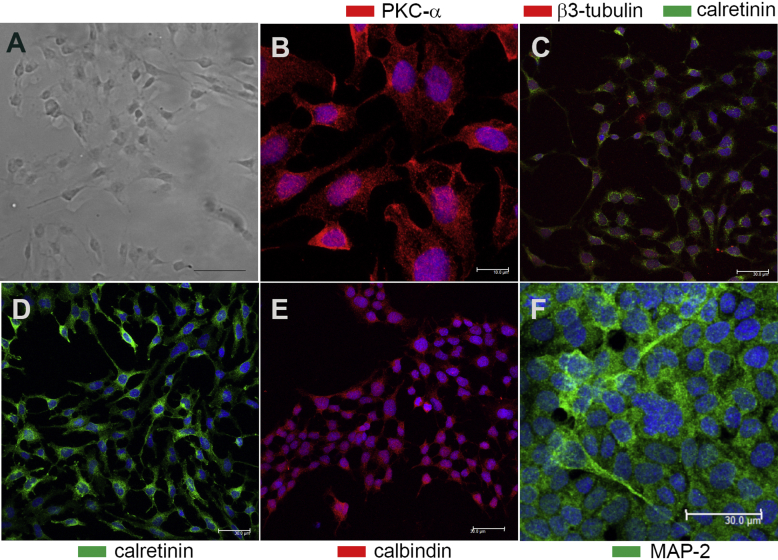
Confocal immunofluorescence confirms the expression of R28 neuronal markers. R28 cultures were probed with a variety of antibodies against specific neuronal markers and then imaged by confocal immunofluorescence microscopy. [A] Brightfield image showing typical morphology of R28 neurons. [B] Expression of protein kinase Cα (red), [C] β3-tubulin (red) and calretinin (green). [D] Calretinin expression alone (green) with [E] calbindin (red) and [F] the microtubule associated protein-2 (green). [B–F] Nuclei are labelled throughout with DAPI (blue) in maximal projections of confocal z-stacks. Scale bar in [A] and [B] corresponds to 40 μm and 10 μm, respectively, while scales in other images [C–F] correspond to 30 μm. (For interpretation of the references to colour in this figure legend, the reader is referred to the web version of this article.)

In order to study dynamic Aβ effects on neuroretinal cells at single-cell resolution we utilized the rat R28 cell-line (Seigel, 1996). The diverse natures of these cultures are comparable to the heterogeneous population of different neuronal cell-types in the mammalian retina (Seigel, 2014, Seigel et al., 2004). R28 cells have been shown to express key neuronal markers including calbindin, protein kinase C (PKC) and MAP-2 by microarray, immunocytochemistry and/or immunoblotting analysis (Bodur and Layer, 2011, Seigel et al., 2004). As we wished to determine whether Aβ could impair neurons by disrupting these markers, we first confirmed their expression by confocal-IF which has not been demonstrated before. Our findings showed the expression of PKC-α (Fig. 2B), β3-tubulin (Fig. 2C), calbindin (Fig. 2E), as well as the microtubule associated protein-2 (MAP-2) (Fig. 2F). We also demonstrated that these neurons expressed calretinin (Fig. 2C, D), which has not been reported in R28 cells previously.

### 3.3 Aβ is rapidly internalised by R28 neuroretinal cells

**Fig. 3:**
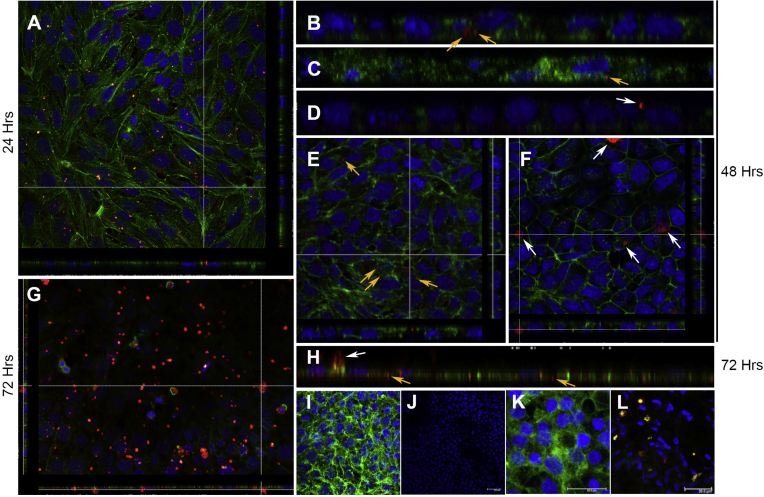
Confocal immunofluorescence reveal Aβ internalisation in R28 cultures. Neurons were treated with either oligomeric Aβ_1-42_ or vehicle, and probed with anti-Aβ 82E1 followed by a fluorescence secondary antibody (red). Representative confocal z-stacks reveal small punctate Aβ signals [A] 24 h and [B] 48 h after exposure, which appeared to be mainly intracellular in distribution (arrows). Abeta (red) in the top-down view [A] appear as yellow within neurons stained with phalloidin (green) with nuclear DAPI in blue. [B] Magnified orthogonal section clearly showing the intracellular localisation of such Aβ particles within neurons (arrows). [C] We also tracked Aβ by direct conjugation with an Alexa Fluor 647 motif. In cultures treated with the directly tagged Aβ (pseudo coloured red), a similar pattern of small punctate fluorescence signals were visible within neurons (arrow). Signals from small internalised Aβ [B, C] were dimmer in intensity compared to brighter signals [D] that appeared to be outside cells (arrow). PKC and nuclear DAPI [in C, D] are in green and blue, respectively. [E, F] Top-down view through confocal z-stacks with corresponding orthogonal section showing; [E] small internalised Aβ (yellow arrows) within cells, and [F] larger Aβ clusters (white arrows) appear to be outside cells 48 h after exposure. Phalloidin and nuclear/DAPI can be seen coloured green and blue, respectively. [G] The propensity of Aβ tagged fluorescence signals (red) to seemingly aggregate outside neurons appear to have increased by 72 h as shown by confocal z-stacks. [H] Magnified orthogonal section at 72 h showing a mixture of small internalised Aβ (yellow arrows) which remained unchanged in size (compare with B-C at earlier time points), vs. larger fluorescence signals from apparently extracellular Aβ (white arrow). [I, J] Maximal intensity projections of representative cultures treated with vehicle control and secondary antibody only (red). Cultures were stained with [I] phalloidin (green) and [J] without phalloidin demonstrates absence of non-specific signals. Nuclear/DAPI appears in blue. [K] Maximal intensity projection showing cultures not treated with tagged Aβ_1-42_-Alexa Fluor 647 (pseudo red) were devoid of any signals. PCK and nuclear/DAPI appear in green and blue, respectively. [L] Cultures treated with Aβ_1-42_-Alexa Fluor 647 (pseudo red) which co-localised with anti-Aβ 82E1 (green) to appear yellow, indicating that all Alexa Fluor tags were positive for Aβ. Nuclear/DAPI appears in blue. Scale bars corresponds to [I] 10 μm, [K] 20 μm and [J, L] 30 μm, respectively. (For interpretation of the references to colour in this figure legend, the reader is referred to the web version of this article.)

R28 cultures were treated with either 1000 nM of oligomeric Aβ_1-42_ or vehicle alone. Neurons were also incubated with Alexa 647-tagged Aβ_1-42_. At 24 and 48 h after incubation, confocal-IF microscopy revealed small, discrete signals corresponding to Aβ which were mainly intracellular in distribution (Fig. 3A–C, E). Signals from such internalised Aβ particles (untagged as well as tagged Aβ) were often quite dim in appearance, and were observed predominantly in the perinuclear region of neurons. By contrast, larger clusters of brighter fluorescent Aβ particles, which were evident by 48 h, appeared to be extracellular (Fig. 3D, F). This trend to aggregate extracellularly continued, and by 72 h appeared as highly visible, large, bright fluorescent clusters (Fig. 3G). A mixture of Aβ particles were therefore observed at 72 h (Fig. 3H); smaller internalised Aβ particles remained unchanged in size from earlier time points, whilst larger Aβ aggregates appeared to have formed extracellularly (Fig. 3G, H). No signals were evident in vehicle treated controls or cultures treated with the secondary antibody only (Fig. 3I, J). Cultures treated with vehicle only in tagged Aβ experiments showed no signals (Fig. 3K); indicating absence of non-specific binding. Importantly, by probing with the anti-82E1 antibody, we confirmed that fluorescent signals in Alexa 647-tagged Aβ_1-42_ treated cultures were specific for Aβ, and that Aβ had not disassociated from its fluorescent motif (Fig. 3L). Aβ appears not to have any discernible effects on the expression/distribution of calbindin, PKC or calretinin in R28 neurons (data not shown).

### 3.4 Aβ transiently impairs the microtubule associated protein-2 (MAP-2)

**Fig. 4:**
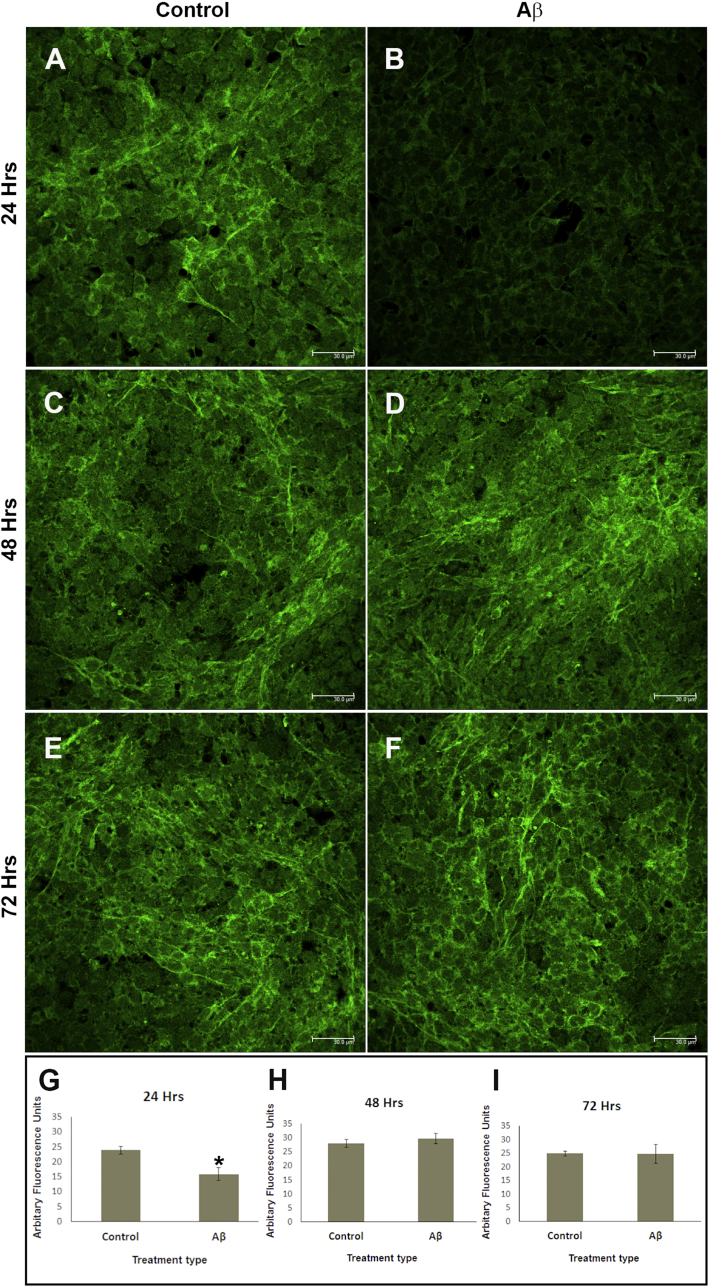
MAP-2 becomes transiently impaired to by Aβ. Neurons were treated with either oligomeric Aβ_1-42_ or vehicle only. Potential effects on MAP-2 were assessed by confocal immunofluorescence microscopy at different time points. [A-B] Representative images show that 24 h after exposure, MAP-2 fluorescence intensity appeared to be considerably diminished in Aβ treated cultures compared to controls. This appeared to be transient as no obvious effects were observed between Aβ vs. vehicle treated cultures at [C, D] 48 h and [E-F] 72 h. Scale bars correspond to 30 μm. MAP-2 fluorescence intensity (green) was quantified blindly in images taken randomly across different time points in at least 3 separate experiments. [G] 24 h after Aβ exposure, average fluorescence values in maximal intensity projections were 15.9, SEM ± 2.1 (n = 6) vs. 23.9, SEM ± 1.29 (n = 4) in vehicle treated cultures. [H] Similar measurements taken 48 h after cells were treated with Aβ were 29.7, SEM ± 1.8 (n = 3) vs. 28.0, SEM ± 1.4 (n = 3) in vehicle only cultures, and [I] after 72 h 24.8, SEM ± 3.4 (n = 3) in Aβ treated vs. 24.9, SEM ± 0.9 (n = 3) in vehicle treated cultures. Error bars represents the standard error of the mean (SEM). Statistical significance (p < 0.05 denoted by *) was evident only at 24 h. (For interpretation of the references to colour in this figure legend, the reader is referred to the web version of this article.)

The exposure of R28 cultures to physiological amounts of oligomeric Aβ_1-42_ (1000 nM) resulted in the downregulation of neuronal MAP-2 after 24 h (Fig. 4A, B). However, no such effects were observed at 48 (Fig. 4C, D) or 72 h (Fig. 4E, F). Quantification of MAP-2 revealed an approximately 33% reduction in total fluorescence intensity (arbitrary fluorescence units or AFU) compared to vehicle treated cultures (Fig. 4G). However, MAP-2 fluorescence levels in Aβ treated neurons had recovered by 48 h, and appeared to be stable afterwards (Fig. 4H, I). Although MAP-2 fluorescence was transiently impaired, its intracellular pattern of distribution remained unaffected (Fig. 4A–F).

### 3.5 Subretinally injected Aβ localises to the neuroretina

**Fig. 5:**
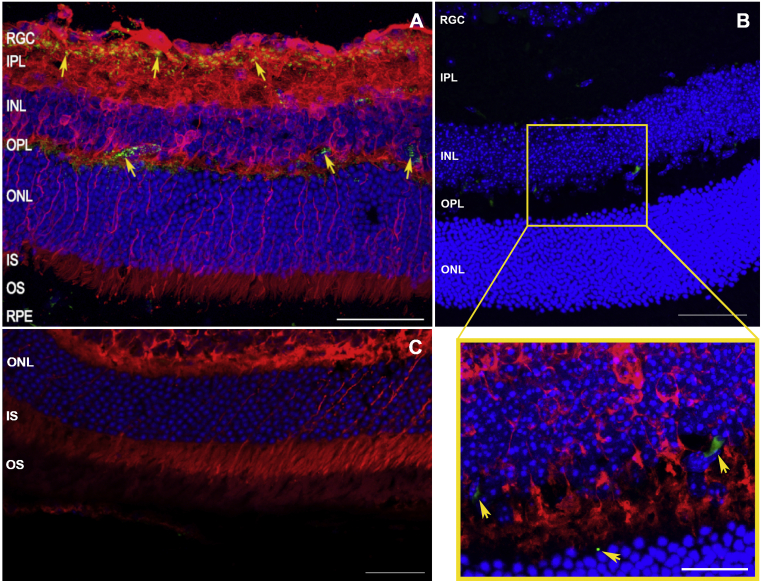
Experimentally introduced Aβ deposit as discrete focal aggregates in the neuroretina. In order to assess whether physiological amounts of Aβ can deposit in living retinas, C57BL/6 mice were subretinally injected with 625 nM of human Aβ_1-42_ or vehicle control. Retinal cross-sections were examined blindly in at least 3 separate animals (n = 10 Aβ injected eyes, and n = 3 vehicle control eyes) by confocal-immunofluorescence after 8 days. Representative images from at least 20 confocal z-stacks consistently show [A-B] Aβ deposits in RGC, inner nuclear and outer plexiform layers (arrows) as visualised by anti-Aβ 82E1 coupled to a secondary fluorescence antibody (green). β3-tubulin and nuclear DPAI appear red and blue, respectively. [B] The red channel (β3-tubulin staining) was removed to highlight focal Aβ deposits (green) within an enlarged section (yellow box), which shows a detailed image of retinal Aβ aggregates (arrows). Scale bars in A, B corresponds to 50 μm, whilst the scale bar in magnified insert is 20 μm [C] Eyes injected with vehicle only lacked Aβ immunoreactivity. β3-tubulin and nuclear DPAI appear red and blue, respectively. The scale bar corresponds to 50 μm. (For interpretation of the references to colour in this figure legend, the reader is referred to the web version of this article.)

Although APP/Aβ immunoreactivity was previously reported in whole retinal mounts, in RGC, the inner nuclear layer and on photoreceptors (Dasari et al., 2011, Dutescu et al., 2009, Guo et al., 2007, Hoh et al., 2010, Kipfer-Kauer et al., 2010, Koronyo-Hamaoui et al., 2011, Wang et al., 2011), we wanted to establish whether a predetermined amount of physiological Aβ_1-42_ was realistically capable of localising to the living retina. To assess if this was possible, ∼2 μl of oligomeric of Aβ_1-42_ (625 nM) was subretinally injected into C57BL/6 wildtype mice to recapitulate the elevated Aβ burden reported in aged and AMD retinas (Ratnayaka et al., 2015). Tissues were analysed away from site of the injection to exclude the possibility of erroneously studying retinal sections with any mechanically induced trauma. Importantly, subretinal injections via the sclera ensured that the needle did not pass through the neuroretina which remained intact and undisturbed throughout the procedure. In this way, we ensured that retinal Aβ immunoreactivity did not arise due to direct contact of the needle with the retina. Use of the anti-Aβ 82E1 antibody excluded the likelihood of APP cross-reactivity, ensuring that signals were only due to the presence of bona fide Aβ deposits. Assessment of retinal cross sections by confocal-immunofluorescence 8 days after injections revealed distinct Aβ deposits in areas corresponding to RGC, inner nuclear and the outer plexiform layers (Fig. 5A, B). Optically enhanced confocal sections showed magnified images of these Aβ aggregates, which appeared as distinct fluorescence signals distributed in the perinuclear region of neurons (Fig. 5B insert). No fluorescence signals from Aβ were observed in vehicle injected eyes (Fig. 5C).

## 4 Discussion

Several laboratories have demonstrated the presence of Aβ aggregates in aged and AMD retinas (Ratnayaka et al., 2015). These studies using histological and confocal-immunofluorescence approaches in wildtype rodent, rabbit, as well as human donor eyes showed Aβ deposits predominantly in the outer retina, focused around the RPE; specifically within drusen, in the RPE-BrM interface and on photoreceptor outer segments (Anderson et al., 2004, Hoh et al., 2010, Isas et al., 2010, Johnson et al., 2002, Luibl et al., 2006). One such study showed that nM amounts of oligomeric Aβ induced a pro-inflammatory environment by elevating interleukin-8 and matrix metalloproteinase-9 in RPE, whilst driving cellular senescence and damaging the RPE barrier function (Cao et al., 2013). Similar findings by other groups revealed that Aβ also activated the complement system to bring about a state of chronic inflammation in subretinal tissues (Catchpole et al., 2013, Koyama et al., 2008, Wang et al., 2008, Wang et al., 2012b, Yoshida et al., 2005). However, retinal neurons are also immunopositive for APP/Aβ (Dasari et al., 2011, Dutescu et al., 2009, Guo et al., 2007, Hoh et al., 2010, Kipfer-Kauer et al., 2010, Koronyo-Hamaoui et al., 2011, Wang et al., 2011), suggesting that Aβ activity is not exclusive to the RPE. Therefore, to fully understand how Aβ could impair the senescent eye, it was important to study whether the neuroretina is also affected by this neurotoxic group of peptides. We therefore set out to address this important question which has thus far garnered only limited attention.

Recent insights into Aβ-mediated pathogenicity showed that oligomeric rather than fibrillar Aβ to be the most toxic and closely associated with AD neuropathology (Benilova et al., 2012, McLean et al., 1999). Although both oligomeric and fibrillar Aβ have been reported surrounding the RPE (Isas et al., 2010, Luibl et al., 2006, Ratnayaka et al., 2015), there is a scarcity of such details with respect to Aβ deposits in the neuroretina itself. Here, we sought to determine the effects of oligomeric Aβ in the retina due to its strong associations with neuronal dysfunction. With regards to Aβ species, both Aβ_1-40_ and Aβ_1-42_ are likely to be present in the retina, as their soluble forms were reported in pico-nano molar range concentrations in bovine vitreous (Prakasam et al., 2010). Our studies focused on oligomeric Aβ_1-42_; as this highly neurotoxic and aggregate-prone Aβ species has been the focus of considerable work in neurodegeneration (Benilova et al., 2012, Soura et al., 2012). As neurons in the central nervous system are known to secrete small soluble Aβ forms (Benilova et al., 2012, McLean et al., 1999), we reasoned that this was also likely to be the case in the retina, particularly as some retinal neurons are immunopositive for APP/Aβ (Dasari et al., 2011, Dutescu et al., 2009, Guo et al., 2007, Koronyo-Hamaoui et al., 2011, Wang et al., 2011). The use of antibodies that cannot distinguish between APP vs. Aβ in past studies (Dasari et al., 2011, Wang et al., 2011) have resulted in some confusion. Nonetheless, APP/Aβ immunoreactivity is likely to correspond to discrete Aβ deposits, at least in some instances (Guo et al., 2007, Kipfer-Kauer et al., 2010, Koronyo-Hamaoui et al., 2011). Given the long term nature of such Aβ deposits, these are probably fibrillar in nature (Benilova et al., 2012, Williams et al., 2010). A dynamic equilibrium is thought to exist between fibrillar Aβ deposits and toxic Aβ oligomers, creating a local spillover of neurotoxic Aβ species in surrounding tissues (Benilova et al., 2012). Hence, retinal Aβ deposits may act as a persistent reservoir for toxic Aβ forms such as oligomers. Neurons of the retina are therefore likely to be exposed to soluble oligomeric Aβ from several sources. Consequently, our experiments sought to recapitulate conditions where neuroretinal cells were exposed to at least one Aβ conformation (oligomeric), species (Aβ_1-42_) and physiological amounts (nM concentrations) that the living retina might realistically be exposed to over decades. As it was important to use oligomeric Aβ, we first investigated the aggregation dynamics of Aβ_1-42_ in our preparations to determine when they were most prevalent. Our negative stain TEM and immunogold labelling experiments consistently showed discrete particles ranging between 15 nm and 65 nm in diameter, which corresponded to the reported size of Aβ oligomers (Williams et al., 2010). These were abundantly visible at ∼1½ h after preparation; the time point at which they were harvested for use in our experiments.

There is considerable debate as to how Aβ might mediate its pathogenicity. Several models have been proposed include carpeting, pore-forming and detergent-like mechanisms by which membrane-damage may occur (Williams and Serpell, 2011). In the current study, we observed that retinal neurons internalised Aβ as early as 24 h after exposure. Our previous work in SH-SY5Y neurons and in cultured hippocampal cells showed that Aβ was internalised via clathrin-positive membrane invaginations (Soura et al., 2012). These findings were consistent with observations that clathrin-mediated mechanisms may at least be partly involved in Aβ internalisation (Song et al., 2011, Wu and Yao, 2009). Although we did not test internalisation mechanisms per se, similar processes may also be involved in the retina. We assessed the capacity of Aβ to enter R28 neurons by two approaches; by using a secondary antibody to probe for untagged Aβ, and by directly tagging Aβ with a fluorescent Alexa Fluor moiety. We reasoned that the former approach was least likely to interfere with conformational arrangements and thus preserve the ‘endogenous’ nature of Aβ oligomers. Nonetheless, both methods consistently demonstrated that Aβ had entered neurons by 24 h. Although we exposed neurons to oligomeric Aβ, once in culture, further experimental manipulation became more difficult. For instance, we cannot regulate the rate of aggregation or the manner in which Aβ may subsequently behave. However, Aβ assembly is known to be influenced by local conditions such as temperature, the presence of reactive oxygen species, metals, cholesterol as well as nearby Aβ deposits (Al-Hilaly et al., 2013, Benilova et al., 2012, Wang et al., 2012a, Williams et al., 2010). Such parameters are likely to impact on how and where Aβ may aggregate in susceptible retinas. We reasoned that Aβ particles that successfully entered neurons were likely to be oligomeric in nature as these are known to be highly penetrative (Benilova et al., 2012, Williams et al., 2011). We observed that the relative size of such internalised Aβ particles did not alter and remained consistently stable for at least 72 h. In contrast, a proportion of oligomers seemed to have undergone further assembly; appearing as large fluorescent clusters on cell surfaces. These appear to be extracellular; their larger size and apparent inability to enter neurons suggesting that they were perhaps no longer oligomeric. An observation consistent with a report that fibrillar Aβ, compared to oligomers, are less liable to enter through membranes (Williams et al., 2010). Insightful as our findings are, these experiments were carried out in R28 neurons, and we cannot rule out the possibility that primary neuroretinal cells may internalise Aβ at different rates. For example, RPE cell-lines and primary RPE cells phagocytose photoreceptor outer segments at different rates (Mazzoni et al., 2014). Genetically and phenotypically however, R28 cells are highly suitable for these studies, as they display the diversity of different neuronal types in the living retina (Seigel, 2014, Seigel et al., 2004). Moreover, these cells do not suffer from problems associated with the widely utilized RGC-5 immortalised cell-line; which was once thought to be a rat retinal ganglion cell-line (Krishnamoorthy et al., 2001) but was recently demonstrated to be a transformed mouse photoreceptor cell-line instead (Krishnamoorthy et al., 2013). This has led to those using the RGC-5 cell-line urging fellow researchers to first establish the expression of specific retinal markers before considering their use as ‘retinal ganglion-like cells’ (Sippl and Tamm, 2014, Van Bergen et al., 2009). Prior to studying possible Aβ effects, we also demonstrated for the first time that R28 neurons express calbindin, PKC-α and MAP-2 by high-resolution confocal microscopy. This confirms their presence reported earlier by microarray, immunocytochemistry and/or immunoblotting approaches (Bodur and Layer, 2011, Seigel et al., 2004). We also showed for the first time that R28 cells express calretinin; a neuronal Ca^2+^-binding protein involved in calcium signalling.

We found that exposure to nM quantities of oligomeric Aβ for 24 h had downregulated MAP-2. The extent of this impairment was considerable, corresponding to ∼33% loss of MAP-2 fluorescence intensity compared to controls. Previous studies of MAP-2 have also used immunofluorescence and immunohistochemistry quantification to gain insights into its behaviour (Kharlamov et al., 2009, Lingwood et al., 2008, Yan et al., 2010). Without further studies, we are unable to comment on potential mechanisms that underlie transient MAP-2 impairment. However, insights may be found in studying interactions between the AD-associated tau protein, which share homology with MAP-2 (Dehmelt and Halpain, 2005), and Aβ (Do et al., 2014). MAP-2 is typically expressed in axons and dendrites of neurons. MAP-2 expression has been reported in anuran (Gabriel et al., 1992), avian (Tucker et al., 1988) and mammalian retinas (Okabe et al., 1989, Tucker and Matus, 1988); specifically in RGC (Okabe et al., 1989, Smith et al., 2008), amacrine cells (Gabriel et al., 1992) as well as in cell bodies and inner segments of photoreceptors (Tucker and Matus, 1988). MAP-2 binds tubulin via multiple domains at its C-terminus to promote the polymerisation of microtubules, whilst its N-terminus contains a projection domain exerting a long-range repulsive action (Dehmelt and Halpain, 2005). MAP-2 upregulation is strongly correlated with stabilising dendrites and is required for activity-dependent stabilisation of new dendritic arbours (Vaillant et al., 2002). Furthermore, MAP-2 is critical to maintaining the spacing between microtubules (Harada et al., 2002, Teng et al., 2001), and protects dendritic microtubules from microtubule-severing enzymes such as katanin that could otherwise destabilise dendrites (Sudo and Baas, 2010). Although MAP-2 activities have not been extensively investigated in the eye, it is likely to carryout similar functions in the retina. MAP-2 is expressed by R28 neurons (Seigel et al., 2004), while MAP-2 immunoreactivity is reported to be a reliable and quantifiable marker of early neuronal injury (Lingwood et al., 2008). Our studies showed that 48 h after Aβ exposure, there were no longer any differences between treated and control cultures, suggesting that either Aβ was unable to disrupt MAP-2 beyond 24 h and/or that adaptive responses to chronic Aβ exposure had restored MAP-2 by then. Such apparently transient Aβ effects have also been reported by others (Benilova et al., 2012, Dal Pra et al., 2011). We also observed that Aβ-mediated damage was specific to MAP-2, as there were no discernible effects on retinal calbindin, PKC or calretinin.

Use of in-vitro neuroretinal cultures was critical in obtaining single-cell readouts via high-resolution imaging, which would not have been possible in whole in-situ retinas. Direct experiments of this nature in living retinas would also pose considerable problems to studying dynamic aspects of Aβ-mediated pathogenicity. Similar issues confront studies into the AD brain, which has resulted in researchers exploiting numerous cell culture models to address fundamental questions. Indeed, we have previously used cultured neurons to demonstrate that dynamic activities of actin underpin extrasynaptic vesicle recycling in the hippocampus (Ratnayaka et al., 2011). An important question that still remained to be clarified was; how likely is Aβ to impair MAP-2 in the living retina? Previous studies have indicated neurons in the RGC and the inner retina to be immunopositive for Aβ (Dutescu et al., 2009, Guo et al., 2007, Kipfer-Kauer et al., 2010). However, several of these studies utilized antibodies that do not necessarily distinguish between Aβ and APP (Dasari et al., 2011, Wang et al., 2011). In order to assess if such an outcome might be realistic, we subretinally injected oligomeric Aβ_1-42_ in nM amounts into wildtype C57BL/6 mice; in proximity to where Aβ synthesis/deposition is reported to be most prevalent (Anderson et al., 2004, Hoh et al., 2010, Isas et al., 2010, Johnson et al., 2002, Luibl et al., 2006). When retinas were analysed after 8 days, distinct areas corresponding to focal Aβ deposits were observed in the RGC, the inner nuclear and outer plexiform layers in addition to subretinal Aβ aggregates. The accumulation of Aβ within these retinal layers supported the realistic possibility that MAP-2 activity in axons/dendrites of RGC, bipolar, horizontal and photoreceptors may be targeted by Aβ. Use of the anti-Aβ 82E1 antibody precluded the possibility of APP cross-reactivity, and demonstrated for the first time that Aβ was indeed capable of accumulating in living retinas after chronic exposure; thus recapitulating the pattern of Aβ deposition reported in native tissues (Dasari et al., 2011, Dutescu et al., 2009, Guo et al., 2007, Hoh et al., 2010, Kipfer-Kauer et al., 2010, Koronyo-Hamaoui et al., 2011, Wang et al., 2011). Immuno-surveillance by migratory retinal microglia/microphages have been shown to engulf retinal Aβ (Hoh et al., 2010). Although we did not specifically probe for microglia/macrophages, the pattern of Aβ deposition observed excludes the likelihood that they originated from such migratory scavengers which display a distinct starburst morphology (Hoh et al., 2010, Walsh et al., 2005). Once introduced subretinally into the living retina, we were also unable to distinguish between oligomeric and fibrillar Aβ. However, based on our understanding of how Aβ behaves in-vitro, under culture conditions and in other studies (Benilova et al., 2012, Williams et al., 2010), we speculate that a majority of oligomeric Aβ_1-42_ had formed in-situ fibrillar retinal deposits. This does not preclude the possibility that some oligomers persisted, as we detected the presence of smaller Aβ forms in-vitro and in cultures at later time points, consistent with the possibility that toxic oligomers could exist in equilibrium around fibrillar Aβ deposits (Benilova et al., 2012).

Although MAP-2 dysregulation had been implicated in AMD retinas, until now there were no insights into how this could occur. For instance, an association of MAP-2 variants were reported in patients with advanced AMD (Zhang et al., 2008), whilst analysis of donor GA AMD retinas revealed MAP-2 labelling in the inner segments of abnormal photoreceptors with neurite sprouts, tortuous axons and abnormally located nuclei (Pow and Sullivan, 2007). However, not all such abnormal neurons were MAP-2 positive. Hence, the authors speculate that MAP-2 expression may be transient and expressed only during neuro-morphogenesis (Pow and Sullivan, 2007) that occurs in post-mitotic neurons (Grandel et al., 2006, Reye et al., 2002, Rich et al., 1997). If Aβ is capable of transiently impairing MAP-2, it may hinder the processes of neuronal plasticity reported in AMD retinas (Sullivan et al., 2007). Our future studies will assess if transient MAP-2 impairment can be visualised or ‘captured’ in-vivo by culling mice at earlier time points. In this way we will be able to draw direct comparisons between in-vitro and in-vivo Aβ effects. Similar Aβ effects on other cytoskeletal components including actin may affect extrasynaptic signalling that also underpins neuronal plasticity (Ratnayaka et al., 2011). These subtle mechanisms may not only contribute to chronic retinal damage, but may also help explain different AMD phenotypes, for instance when primary photoreceptor pathology is observed without apparent damage to the underlying RPE (Bird et al., 2014). Such a possibility is supported by studies showing that Aβ is capable of inducing a variety of retinal pathophysiologies, for instance when intravitreal Aβ injections in rodents resulted in retinal inflammation (Howlett et al., 2011, Walsh et al., 2005) - a well-documented feature in AMD retinas (Kauppinen et al., 2016, Khandhadia et al., 2012). The therapeutic benefits of reducing retinal Aβ was highlighted in an elegant study where visual abnormalities in a human ApoE4 knock-in mouse that was aged and fed a high fat diet were abolished by anti-Aβ immunotherapy (Ding et al., 2011). Furthermore, reducing Aβ formation, clearance of Aβ deposition as well as inhibiting Aβ aggregation in a mouse study of experimentally-induced glaucoma not only revealed the role of Aβ in other retinal disorders, but also highlighted the therapeutic benefits of inhibiting its retinal activities (Guo et al., 2007). Our findings show how Aβ is rapidly internalised to accumulate within retinal neurons, whilst a proportion of extracellular Aβ appear to assemble, perhaps forming focal Aβ deposits observed in aged/AMD retinas. Specific Aβ-mediated damage to MAP-2 in cultured neurons as well as Aβ deposition in living retinas suggests that it has the capacity to impair neuronal modelling and plasticity. Related questions such whether other Aβ species may be involved, what effects longer Aβ exposures may produce, and what other cytoskeletal elements may be targeted remains the subject of further studies in our laboratory.
